# The Evolution of Self-Gravitating Radiating Geodesic Fluid Spheres Instantaneously Admitting a Time-like Killing †

**DOI:** 10.3390/e28060684

**Published:** 2026-06-13

**Authors:** Luis Herrera, Alicia Di Prisco

**Affiliations:** 1 Instituto Universitario de Física Fundamental y Matemáticas, Universidad de Salamanca, 37007 Salamanca, Spain; 2 Escuela de Física, Facultad de Ciencias, Universidad Central de Venezuela, Caracas 1050, Venezuela; alicia.diprisco@ucv.ve

**Keywords:** relativistic fluids, interior solutions, spherically symmetric sources, 04.40.-b, 04.40.Nr, 04.40.Dg

## Abstract

In this study, we analyze the effects produced by the sudden appearance of a time-like Killing vector (TKV) on the evolution of radiating geodesic fluids. This is achieved by resorting to the concept of an asymmetry factor, which allows the instantaneous appearance of a given symmetry for a given value of a time-like coordinate. All relevant physical variables are analyzed in detail, showing the imprints of the emergence of TKV at some point in the evolution. The main lesson that can be extracted from the obtained results is that the sudden appearance of a TKV, even for an infinitesimal period of time, drastically changes the subsequent evolution of the system. Of particular relevance is the appearance of a thermal effect linking the asymmetry factor and the relaxation time. The potential applications of these results in the study of gravitational collapse are discussed at the end of the paper.

## 1. Introduction

In a recent paper [1], we introduced the concept of the asymmetry factor, which is a scalar function allowing one to switch on (or off) a given symmetry for any value of the time-like coordinate. More specifically, in [1], we considered the case of a conformal Killing vector, and we looked for the imprints that the instantaneous appearance of such a symmetry produces on the evolution of the fluid. In this work, we want to apply a similar scheme to a case where the involved symmetry corresponds to a time-like Killing vector field, and the matter content corresponds to geodesic dissipative fluids. This type of fluid has been shown to be very useful in the study of gravitational collapse (see [2,3,4,5,6,7,8] and the references therein).

The main reason to consider a TKV as the instantaneously appearing symmetry is that radiating spherically symmetric fluid distributions, in general, do not admit a TKV; therefore, we wonder what influence this might have on the evolution of the system. In other words, we wanted to find out how to describe the evolution of the system in terms of the factor measuring deviations from the time invariance (the asymmetry factor).

Thus, we shall assume geodesic spherically symmetric fluid distributions that, at some point of their evolution, admit a TKV. The (dis)appearance of the TKV is controlled by a tensor defined through the asymmetry factor. As we will see below, the sudden appearance of the TKV, even during an infinitesimal small interval of time, has a remarkable influence on all physical variables.

All relevant variables characterizing the obtained model are analyzed in detail, and the influence of the appearance of the TKV on the behavior of the complexity factor, the anisotropic factor, the luminosity, the temperature and the redshift is emphasized.

The method and the variables required for describing the evolution of spherically symmetric fluid distributions are presented in the next section. In Section 3, we introduce the concept of an asymmetry factor for a time-like isometry, and express the metric variables in terms of such a factor. A specific model is analyzed in Section 4, and the obtained results are discussed in Section 5. Finally, Appendix A is included.

## 2. Fundamental Variables and Relevant Equations

In this section, we introduce all variables and definitions, allowing for a full description of the structure and evolution of geodesic, spherically symmetric dissipative fluids. For more details, see [9].

For spherically symmetric distributions of evolving geodesic fluids, bounded by a spherical surface Σ, the general interior metric in comoving coordinates reads

(1) $$ds^{2}=-dt^{2}+B^{2}dr^{2}+R^{2}\left(d\theta^{2}+\sin^{2}\theta d\phi^{2}\right),$$

where *B* and *R* are functions of *t* and *r* and are assumed positive. Observe that *B* is dimensionless, whereas *R* has the same dimension as *r*.

The matter content consists of an anisotropic fluid (with principal stresses unequal) undergoing dissipation in the form of heat flow (diffusion approximation).

Thus, the energy–momentum tensor $T_{\alpha \beta }$ may be written as

(2) $$\begin{array}{ccc} T_{\alpha \beta } & = & \left(\mu +P_{⊥}\right)V_{\alpha }V_{\beta }+P_{⊥}g_{\alpha \beta }+\left(P_{r}-P_{⊥}\right)K_{\alpha }K_{\beta }\\ & + & q_{\alpha }V_{\beta }+V_{\alpha }q_{\beta }, \end{array}$$

or

(3) $$T_{\alpha \beta }=\mu V_{\alpha }V_{\beta }+Ph_{\alpha \beta }+\Pi_{\alpha \beta }+q\left(V_{\alpha }K_{\beta }+K_{\alpha }V_{\beta }\right),$$

where μ is the energy density, $P_{r}$ is the radial pressure, $P_{⊥}$ is the tangential pressure, $q^{\alpha }$ is the heat flux, $V^{\alpha }$ is the four-velocity of the fluid, and $K^{\alpha }$ is a unit four-vector along the radial direction, and

(4) $$\begin{array}{c} V^{\alpha }V_{\alpha }=-1,\;\;V^{\alpha }q_{\alpha }=0,\;\;K^{\alpha }K_{\alpha }=1,\;\;K^{\alpha }V_{\alpha }=0. \end{array}$$

$$P=\frac{P_{r}+2P_{⊥}}{3},\;h_{\alpha \beta }=g_{\alpha \beta }+V_{\alpha }V_{\beta },$$

$$\Pi_{\alpha \beta }=\Pi \left(K_{\alpha }K_{\beta }-\frac{1}{3}h_{\alpha \beta }\right),\;\Pi =P_{r}-P_{⊥}.$$

(5) $$\begin{array}{ccc} V^{\alpha } & = & \delta_{0}^{\alpha },\;\;q^{\alpha }=qK^{\alpha },\;\;K^{\alpha }=B^{-1}\delta_{1}^{\alpha }, \end{array}$$

where *q* is a function of *t* and *r*.

The Einstein equations for (1) and (3) are explicitly written in Appendix A.

The expansion Θ of the fluid is given by

(6) $$\Theta ={V^{\alpha }}_{;\alpha },$$

and its shear $\sigma_{\alpha \beta }$ is given by

(7) $$\sigma_{\alpha \beta }=V_{\left(\alpha ;\beta \right)}+a_{(\alpha }V_{\beta )}-\frac{1}{3}\Theta h_{\alpha \beta }.$$

From (6), we have for the expansion

(8) $$\Theta =\left(\frac{\dot{B}}{B}+2\frac{\dot{R}}{R}\right),$$

where the prime stands for *r* differentiation and the dot stands for differentiation with respect to *t*.

The shear tensor (7) has only one independent non-zero component, and may be written as

(9) $$\sigma_{\alpha \beta }=\sigma \left(K_{\alpha }K_{\beta }-\frac{h_{\alpha \beta }}{3}\right),$$

with

(10) $$\sigma =\left(\frac{\dot{B}}{B}-\frac{\dot{R}}{R}\right).$$

Next, the mass function m(t,r) reads [10,11]

(11) $$m=\frac{R^{3}}{2}{R_{23}}^{23}=\frac{R}{2}\left[{\dot{R}}^{2}-{\left(\frac{R^{\prime }}{B}\right)}^{2}+1\right].$$

Introducing the proper time-derivative $D_{T}$ given by

(12) $$D_{T}=\frac{\partial }{\partial t},$$

We can define the areal velocity *U* as the variation in the areal radius with respect to proper time, i.e.,

(13) $$U=D_{T}R,$$

where *R* defines the areal radius of a spherical surface inside the fluid distribution (as measured from its area).

Then, (11) can be rewritten as

(14) $$E\equiv \frac{R^{\prime }}{B}={\left(1+U^{2}-\frac{2m}{R}\right)}^{1/2}.$$

Using (14), we can express (A6) as

(15) $$4\pi q=E\left[\frac{1}{3}D_{R}\left(\Theta -\sigma \right)-\frac{\sigma }{R}\right],$$

where $D_{R}$ denotes the proper radial derivative,

(16) $$D_{R}=\frac{1}{R^{\prime }}\frac{\partial }{\partial r}.$$

### 2.1. The Complexity Factor

The idea of complexity in different branches of science has a long and venerable history (see [12] and references therein). Some years ago, important advances on this issue were achieved by López-Ruiz and collaborators within the context of statistical physics [13,14,15,16]. This approach was later employed by some authors to define complexity for self-gravitating systems (see [17] and references therein). However, such a definition suffers from some flaws, which motivated the search for an alternative definition of complexity for self-gravitating systems. This search led to the definition proposed in [9,18], which is based on the concept of complexity factor.

This is a scalar function measuring the degree of complexity in a fluid distribution [9,18]. Mathematically, it is the scalar defining the trace-free part of the electric Riemann tensor ($Y_{TF}$) [19].

Thus, the tensor $Y_{\alpha \beta }$ defining the electric part of the Riemann tensor given by

(17) $$Y_{\alpha \beta }=R_{\alpha \gamma \beta \delta }V^{\gamma }V^{\delta },$$

may be written as

(18) $$\begin{array}{c} Y_{\alpha \beta }=\frac{1}{3}Y_{T}h_{\alpha \beta }+Y_{TF}\left(K_{\alpha }K_{\beta }-\frac{1}{3}h_{\alpha \beta }\right). \end{array}$$

The scalar $Y_{TF}$ is identified with the complexity factor and may be written in terms of the anisotropy of pressure, the density inhomogeneity and the dissipative variables as follows (see [9,18,19] for details):

(19) $$Y_{TF}=-8\pi \Pi +\frac{4\pi }{R^{3}}\int_{0}^{r}R^{3}\left(D_{R}\mu -3q\frac{U}{RE}\right)R^{\prime }dr.$$

In terms of the metric functions, the scalar $Y_{TF}$ reads

(20) $$\begin{array}{c} Y_{TF}=\frac{\ddot{R}}{R}-\frac{\ddot{B}}{B}. \end{array}$$

### 2.2. The Exterior Spacetime and Junction Conditions

For a smooth matching of (1) with Vaidya on the boundary surface of the fluid distribution, we have to assume the Darmois conditions [20].

Thus, outside Σ, we assume that we have the Vaidya spacetime described by

(21) $$ds^{2}=-\left[1-\frac{2M(v)}{\rho }\right]dv^{2}-2d\rho dv+\rho^{2}\left(d\theta^{2}+\sin^{2}\theta d\phi^{2}\right),$$

where M(v) denotes the total mass, *v* is the retarded time, and ρ is a null coordinate.

The matching of the full non-adiabatic sphere (including viscosity) to the Vaidya spacetime, on the surface $r=r_{\Sigma }=$ constant, was discussed in [21]. It implies the following:

- From the continuity of the first differential form,
  (22) $$dt\overset{\Sigma }{=}dv\left(1-\frac{2M(v)}{\rho }\right)\overset{\Sigma }{=}d\tau ,$$
  (23) $$R\overset{\Sigma }{=}\rho \left(v\right),$$
  and
  (24) $${\left(\frac{dv}{d\tau }\right)}^{-2}\overset{\Sigma }{=}\left(1-\frac{2m}{\rho }+2\frac{d\rho }{dv}\right),$$
  where τ denotes the proper time measured on Σ, and $\overset{\Sigma }{=}$ means that both sides of the equation are evaluated on Σ.
- From the continuity of the second differential form
  (25) $$m\left(t,r\right)\overset{\Sigma }{=}M\left(v\right),$$
  (26) $$q\overset{\Sigma }{=}P_{r}.$$

Also, we have

(27) $$q\overset{\Sigma }{=}\frac{L}{4\pi \rho^{2}},$$

where $L_{\Sigma }$ denotes the total luminosity of the sphere as measured on its surface and is given by

(28) $$L\overset{\Sigma }{=}L_{\infty }{\left(1-\frac{2m}{\rho }+2\frac{d\rho }{dv}\right)}^{-1},$$

and where

(29) $$L_{\infty }=-\frac{dM}{dv}\overset{\Sigma }{=}-\left[\frac{dm}{dt}\frac{dt}{d\tau }{\left(\frac{dv}{d\tau }\right)}^{-1}\right]$$

is the total luminosity measured by an observer at rest at infinity, which is defined by

(30) $$L_{\infty }=4\pi P_{r\Sigma }{\left[R^{2}{\left(\frac{R^{\prime }}{B}+\dot{R}\right)}^{2}\right]}_{\Sigma }.$$

The boundary redshift $z_{\Sigma }$ is given by

(31) $$\frac{dv}{d\tau }\overset{\Sigma }{=}1+z,$$

with

(32) $$\frac{dv}{d\tau }\overset{\Sigma }{=}{\left(\frac{R^{\prime }}{B}+\dot{R}\right)}^{-1}.$$

When Darmois conditions [20] are not satisfied, the boundary surface is a thin shell and we have to assume the Israel conditions [22].

### 2.3. The Transport Equation

For obtaining an expression for the temperature, we shall resort to the transport equation proposed in the context of the Israel–Stewart theory [23,24,25], which for a geodesic fluid reads

(33) $$\begin{array}{c} \tilde{\tau }h^{\alpha \beta }V^{\gamma }q_{\beta ;\gamma }+q^{\alpha }=-\kappa h^{\alpha \beta }T_{,\beta }-\frac{1}{2}\kappa T^{2}{\left(\frac{\tilde{\tau }V^{\beta }}{\kappa T^{2}}\right)}_{;\beta }q^{\alpha }, \end{array}$$

where κ denotes the thermal conductivity, and *T* and $\tilde{\tau }$ denote temperature and relaxation time, respectively. We recall that the relaxation time is the time taken by the system to return to the steady state after it has been suddenly removed from it.

In the spherically symmetric case under consideration, the transport equation has only one independent component, which may be obtained from (33) by contracting with the unit spacelike vector $K^{\alpha }$; through this, we get

(34) $$\tilde{\tau }V^{\alpha }q_{,\alpha }+q=-\kappa K^{\alpha }T_{,\alpha }-\frac{1}{2}\kappa T^{2}{\left(\frac{\tilde{\tau }V^{\alpha }}{\kappa T^{2}}\right)}_{;\alpha }q.$$

In some cases (when the last term in (33) may be neglected [26]), it is possible to simplify the transport equation, producing

(35) $$\tilde{\tau }V^{\alpha }q_{,\alpha }+q=-\kappa K^{\alpha }T_{,\alpha }.$$

## 3. The Asymmetry Factor for Isometries

Spacetimes admitting a TKV satisfy the equation

(36) $$L_{\chi }g_{\alpha \beta }=0,$$

where $L_{\chi }$ denotes the Lie derivative with respect to the vector field χ, which in our case is time-like, and has the form

(37) $$\chi =\partial_{t}.$$

In this work, we shall consider fluid distributions which in general do not admit a TKV, except at a given moment of their evolution.

Thus, let us consider

(38) $$L_{\chi }g_{\alpha \beta }=H_{\alpha \beta },$$

implying

(39) $$\chi^{\rho }\partial_{\rho }g_{\alpha \beta }+g_{\alpha \rho }\partial_{\beta }\chi^{\rho }+g_{\beta \rho }\partial_{\alpha }\chi^{\rho }=H_{\alpha \beta },$$

where $H_{\alpha \beta }$ is (so far) an arbitrary symmetric tensor. Obviously, the vector χ defines a TKV if $H_{\alpha \beta }=0$.

Using (37), one obtains for (1) $H_{00}=H_{01}=H_{02}=H_{03}=H_{13}=H_{12}=H_{23}=0$.

Next, let us write the tensor $H_{\alpha \beta }$ in the form

(40) $$H_{\alpha \beta }=H_{T}\frac{g_{\alpha \beta }}{4}+H_{TF}\left(K_{\alpha }K_{\beta }-\frac{1}{3}h_{\alpha \beta }\right),$$

where $H_{T}$ and $H_{TF}$ are two dimensionless scalar functions depending only on *t*.

Then, we obtain from the above

(41) $$H_{T}=0,$$

(42) $$\dot{B}=\frac{\alpha H_{TF}B}{3},$$

(43) $$\dot{R}=-\frac{\alpha H_{TF}R}{6},$$

where α is a unit constant with dimensions $\frac{1}{length}$.

The scalar function $H_{TF}$ is the asymmetry factor. It enables us to switch on (off) the isometry at any given point of the evolution.

Using the above results, we may write the metric functions as

(44) $$A=1,\;B=g\left(r\right)e^{\frac{\alpha }{3}\int H_{TF}dt},\;\alpha R=h\left(r\right)e^{-\frac{\alpha }{6}\int H_{TF}dt},$$

where *g* and *h* are arbitrary dimensionless functions.

Using these results, we shall build up our model.

At this point, we should notice that if we assume the fluid to be non-dissipative, then feeding back (44) into (A3), we see that q≠0.

Thus, the non–dissipative case cannot be analyzed by means of the asymmetry factor scheme for a TKV and geodesic fluids.

## 4. The Model

Based on the above comments, we are constrained to the dissipative case exclusively.

Using the form (40) of the tensor $H_{\alpha \beta }$, and assuming for $H_{TF}$

(45) $$H_{TF}=\alpha \left(t-t_{0}\right),$$

we obtain

(46) $$A=1\;B=g\left(r\right)e^{y^{2}/6}\;\alpha R=h\left(r\right)e^{-y^{2}/12},$$

where $y\equiv \alpha \left(t-t_{0}\right)=H_{TF}$.

In this case, any possible model is determined up to two functions of *r*.

In order to specify our model, we shall proceed as follows.

Let us introduce the dimensionless variable

(47) $$x\equiv \frac{r}{r_{\Sigma }},$$

and choose for the free parameters the value

(48) $$\alpha r_{\Sigma }=1.$$

Based on the above, the physical variables read

(49) $$\begin{array}{ccc} 8\pi \mu /\alpha^{2} & = & -\frac{y^{2}}{12}-\left[\frac{2h_{,xx}}{h}+{\left(\frac{h_{,x}}{h}\right)}^{2}-\frac{2g_{,x}h_{x}}{gh}\right]\frac{e^{-y^{2}/3}}{g^{2}}\\ & + & \frac{e^{y^{2}/6}}{h^{2}}, \end{array}$$

(50) $$\begin{array}{c} 8\pi q/\alpha^{2}=-\frac{e^{-y^{2}/6}yh_{,x}}{gh}, \end{array}$$

(51) $$\begin{array}{c} 8\pi P_{r}/\alpha^{2}=\frac{1}{3}-\frac{y^{2}}{12}+\frac{e^{-y^{2}/3}h_{,x}^{2}}{g^{2}h^{2}}-\frac{e^{y^{2}/6}}{h^{2}}, \end{array}$$

(52) $$8\pi P_{⊥}/\alpha^{2}=-\frac{1}{6}\left(1+\frac{y^{2}}{2}\right)+\frac{e^{-y^{2}/3}}{g^{2}}\left(\frac{h_{,xx}}{h}-\frac{h_{,x}g_{,x}}{hg}\right),$$

Also, from (28) and (30), we obtain

(53) $$L_{\Sigma }\overset{\Sigma }{=}-\frac{yhh_{,x}e^{-y^{2}/3}}{2g},$$

and

(54) $$\begin{array}{c} L_{\infty }\overset{\Sigma }{=}L_{\Sigma }e^{-y^{2}/2}{\left(\frac{h_{,x}}{g}-\frac{yhe^{y^{2}/6}}{6}\right)}^{2}, \end{array}$$

whereas the expressions for the complexity factor and the redshift read

(55) $$Y_{TF}/\alpha^{2}=-\frac{1}{2}\left(\frac{y^{2}}{6}+1\right),$$

and

(56) $$z\overset{\Sigma }{=}\frac{6ge^{y^{2}/4}}{6h_{,x}-hyge^{y^{2}/6}}-1.$$

Finally, using the truncated version of the transport Equations (35) and (60), we may obtain an expression for the temperature. It reads

(57) $$\begin{array}{c} \tilde{T}=\frac{1}{8\pi }\left[y-\tau^{*}\left(\frac{y^{2}}{3}-1\right)\right]\int \frac{h_{,x}}{h}dx+{\tilde{T}}_{0}\left(y\right), \end{array}$$

where $\tau^{*}=\alpha \tilde{\tau }$ and $\tilde{T}=\frac{\kappa T}{\alpha }$.

To completely determine the model, we still need to specify the functions *h* and *g*.

For simplicity, we choose

(58) g(r)=h(r)=αr=x.

From the above restrictions, the physical variables become

(59) $$\begin{array}{c} 8\pi \mu /\alpha^{2}=-\frac{y^{2}}{12}+\left(\frac{1}{x^{2}}+e^{y^{2}/2}\right)\frac{e^{-y^{2}/3}}{x^{2}}, \end{array}$$

(60) $$\begin{array}{c} 8\pi q/\alpha^{2}=-\frac{ye^{-y^{2}/6}}{x^{2}}, \end{array}$$

(61) $$\begin{array}{c} 8\pi P_{r}/\alpha^{2}=\frac{1}{3}-\frac{y^{2}}{12}+\frac{e^{-y^{2}/3}}{x^{4}}-\frac{e^{y^{2}/6}}{x^{2}}, \end{array}$$

(62) $$\begin{array}{c} 8\pi P_{⊥}/\alpha^{2}=-\frac{1}{6}-\frac{y^{2}}{12}-\frac{e^{-y^{2}/3}}{x^{4}}, \end{array}$$

and from (28) and (30), we obtain

(63) $$L_{\Sigma }=-\frac{ye^{-y^{2}/3}}{2},$$

and

(64) $$\begin{array}{c} L_{\infty }=L_{\Sigma }e^{y^{2}/2}{\left(1-\frac{ye^{y^{2}/6}}{6}\right)}^{2}, \end{array}$$

whereas the expressions for the complexity factor and the redshift read

(65) $$Y_{TF}/\alpha^{2}=-\frac{1}{2}\left(\frac{y^{2}}{6}+1\right),$$

and

(66) $$z_{\Sigma }=\frac{6e^{y^{2}/4}}{6-ye^{y^{2}/6}}-1.$$

Finally, the expression for $\tilde{T}$ becomes

(67) $$\begin{array}{c} \tilde{T}=\frac{\ln x}{8\pi }\left[y-\tau^{*}\left(\frac{y^{2}}{3}-1\right)\right]+{\tilde{T}}_{0}\left(y\right), \end{array}$$

or

(68) $$\begin{array}{c} T^{*}=\frac{\ln x}{8\pi }\left[y-\tau^{*}\left(\frac{y^{2}}{3}-1\right)\right], \end{array}$$

where $T^{*}=\tilde{T}-{\tilde{T}}_{\Sigma }$. This choice of variable ($T^{*}$) allows us to skip the adoption of a specific value for ${\tilde{T}}_{\Sigma }$.

Two comments are in order at this point

- As follows from (10) and (46), the shear of our model is necessarily non-vanishing.
- The expansion scalar of our model vanishes, and we recall that the expansion-free condition necessarily implies the appearance of a cavity around the center [27]. Accordingly, the fact that the physical variables exhibit a singularity at r=0 does not represent a problem for the interpretation of the model. In other words, the fluid under consideration is restricted to values of *x* within the interval $\left[x_{min}>0,1\right]$, where $x_{min}$ is the value of *x* corresponding to the boundary of the cavity.

**Figure 1:**
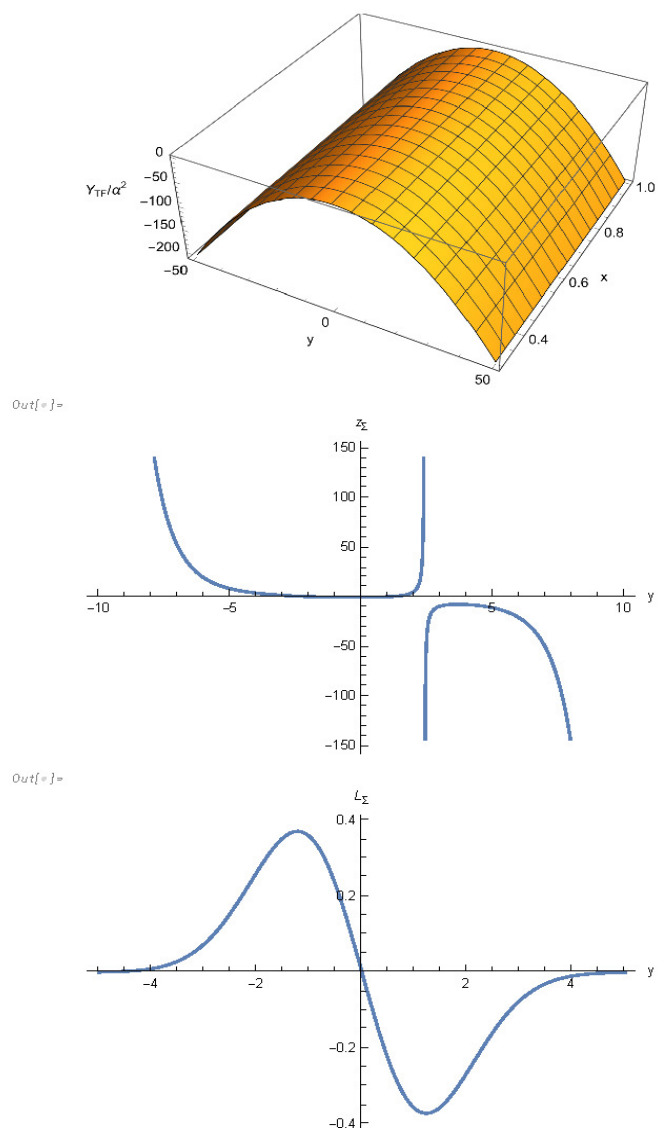
$Y_{TF}/\alpha^{2}$ as function of *y* and *x* in the interval (y,−50,50), (x,0.3,1), 1; $z_{\Sigma }$ in the interval (y,−10,10); $L_{\Sigma }$ in the interval (y,−5,5).

**Figure 2:**
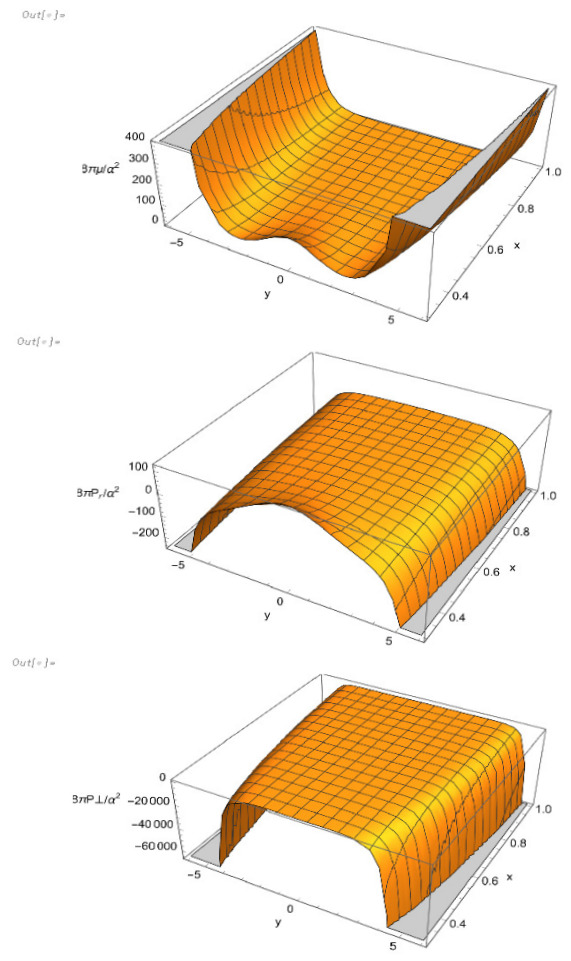
$8\pi \mu /\alpha^{2}$, $8\pi P_{r}/\alpha^{2}$, $8\pi P_{⊥}/\alpha^{2}$ as functions of *y* and *x* in the interval (y,−6,6), (x,0.3,1).

**Figure 3:**
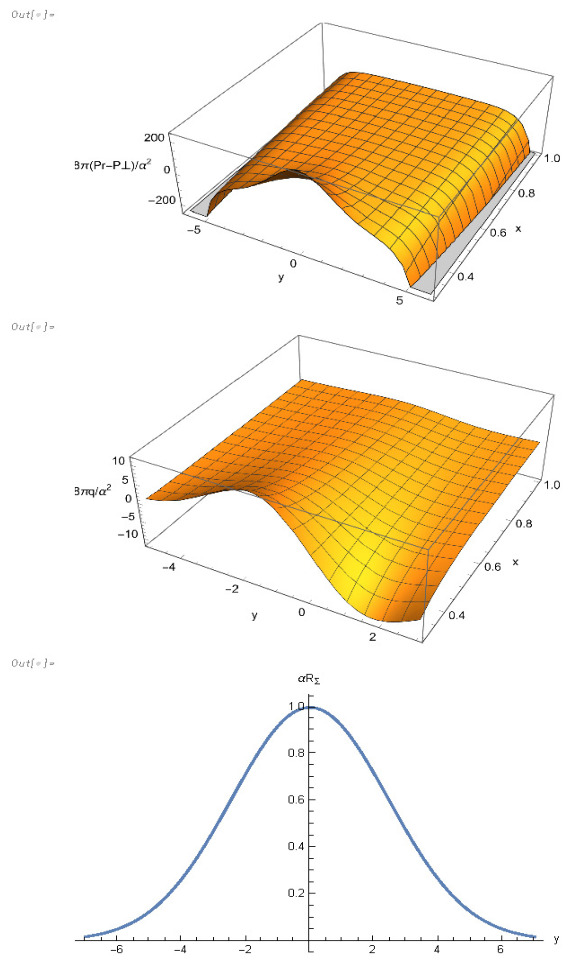
$8\pi \left(P_{r}-P_{⊥}\right)/\alpha^{2}$ as function of *y* and *x* in the interval (y,−6,6), (x,0.3,1); $8\pi q/\alpha^{2}$ as function of *x* and *y* in the interval (y,−5,3), (x,0.3,1) and $\alpha R_{\Sigma }$ in the interval (y,−7,7).

**Figure 4:**
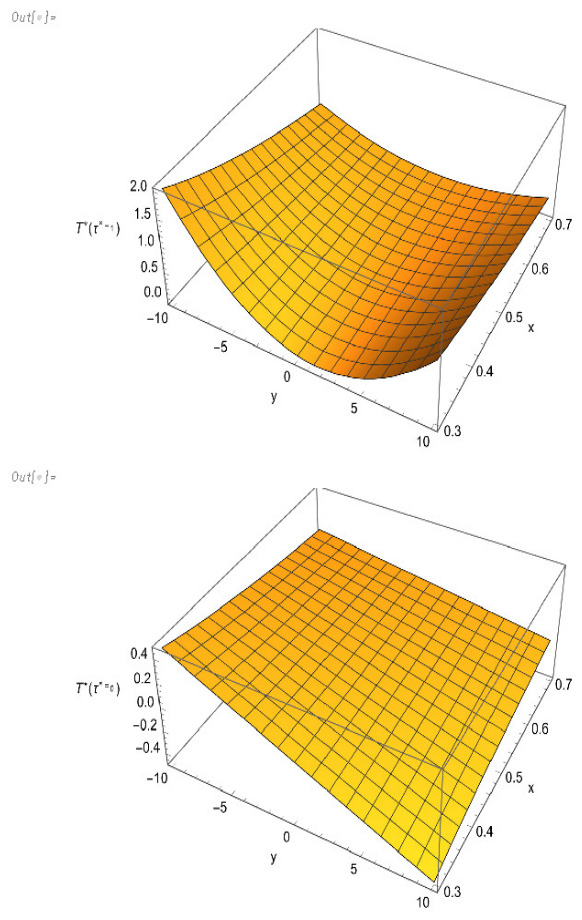
$T^{*}$ as function of *y* and *x* in the interval (y,−10,10), (x,0.3,0.7) for $\tau^{*}=1$ and $\tau^{*}=0$.

To highlight the effects derived from the appearance of the TKV on the evolution of the fluid we plot $Y_{TF}/\alpha^{2}$, $z_{\Sigma }$ and $L_{\Sigma }$ in Figure 1. The dimensionless physical variables $8\pi \mu /\alpha^{2},\;8\pi P_{r}/\alpha^{2},\;8\pi P_{⊥}/\alpha^{2}$ are plotted in Figure 2, whereas $8\pi \left(P_{r}-P_{⊥}\right)/\alpha^{2},\;8\pi q/\alpha^{2}$ and $\alpha R_{\Sigma }$ are depicted in Figure 3. Finally, in Figure 4, we plot the variable $T^{*}$ for two different values of $\tau^{*}$ (1 and 0).

All these results will be discussed in the following section.

## 5. Discussion

The problem of gravitational collapse is one of the most important problems that astrophysicists have to face [28,29,30]. For decades, many researchers have devoted their work to elucidating some of the many questions raised by this issue. Numerical and analytical methods have been applied in such endeavors, each of which present advantages and disadvantages.

In this work, we have adopted an analytical approach based on the assumption that at some point of the evolution, the system momentarily admits a TKV.

Our main purpose was to exhibit the effects of an instantaneous appearance of a TKV on the evolution of relativistic self-gravitating fluids. This was achieved by means of the concept of asymmetry factor introduced in [1], which allows us to turn on (or off) a TKV at any given point of the evolution.

Our model was assumed to be dissipative. Further specifications of this model are related to the specific choice of the two functions of *r*, up to which the general model is defined, and the choice of the form for the asymmetry factor $H_{TF}$; such choices obey to a simplicity criterium.

Under the conditions above, we obtain the model described by (59)–(68). It represents an expanding fluid sphere for y<0 reaching a maximum at y=0 (the moment of the appearance of the TKV), contracting from there on, as shown in the graphic of $\alpha R_{\Sigma }$ in Figure 3.

Since Darmois conditions are not satisfied on the boundary surface of the fluid distribution ($P_{r\Sigma }\neq q_{\Sigma }$), we have to assume that the boundary is a thin shell. At this point, the following comments are in order:

- The presented model was not conceived to describe any specific physical scenario, but just to bring out the effects produced by the sudden appearance of a TKV. Therefore, neither the presence of a thin shell, nor the violation of some energy conditions, are, at this point, relevant for the discussion. All this having being said, it should be clear that the appearance of a thin shell implies that, according to the Israel approach [22], the boundary is endowed with some specific material properties (surface energy-density and tension) whose role in the analysis of our model has not been taken into account. It goes without saying that in a model intended to describe a specific astrophysical object, the dynamics of the shell might be a relevant factor and its role and the nature of its physical origin should be included in the discussion. A similar comment applies to any eventual violation of energy conditions.
- As mentioned before, the choice (45) of the asymmetry factor is justified by its simplicity. However, if we assume that H(t) is a well-behaving function, we may expand it in a power series of $t-t_{0}$, implying that, at least for values of *t* not far from $t_{0}$, the above choice is likely to describe the evolution quite well. Notwithstanding this, it is obvious that H(t) is a degree of freedom of the system, and different choices may be invoked to describe different scenarios of physical interest. Thus, for example, one might propose a Gaussian function, which would make the symmetry appear and then disappear smoothly (we thank one of the referees for this suggestion).
- Our model was obtained under the assumption (58) for the functions h(r) and g(r), which as mentioned before, was adopted for its simplicity, and leads to a particularly simple form of the metric functions in addition to violations of some energy conditions as well as the appearance of thin shells. Although such a choice allows us to bring out the effects of the sudden appearance (or disappearance) of the TKV, it goes without saying that for the treatment of any specific astrophysical scenario, the choice of these functions must be dictated by the very nature of the problem under consideration.

Some remarkable characteristics of this model, illustrated by Figure 1, Figure 2, Figure 3 and Figure 4, deserve to be emphasized:

- The complexity factor presents a local minimum (of its absolute value) at y=0. The existence of a stationary value of the complexity factor at y=0 (minimum or maximum) also appears in the case of a conformal Killing vector (CKV) analyzed in [1], suggesting that a possible model independent link exists between complexity and symmetry.
- The anisotropic factor presents a maximum at y=0. This is also observed in the case of the CKV, suggesting a model-independent link between the anisotropy factor and symmetry.
- There is a discontinuity in the surface redshift at around y≈2.36. Furthermore, the sign of $z_{\Sigma }$ changes along the evolution (from redshift to blueshift). This very peculiar behavior of the redshift is probably highly model-dependent. Notwithstanding this, it suggests the possibility of observational support for the approach presented here.
- Particular attention should be given to the behavior of $T^{*}$ depicted in Figure 4. In the upper graphic, $T^{*}$ is plotted for $\tau^{*}=1$, and it was found that the same behavior (qualitatively) is observed for any value of $\tau^{*}\neq 0$; namely, $T^{*}$ decreases as *y* approaches 0, increasing afterward. However, if we assume that the relaxation time vanishes, then as depicted in the corresponding graphic, $T^{*}$ monotonically decreases after passing through y=0 (notice that, unlike the “physical” temperature *T*, the variable $T^{*}$ may be negative). In other words, the increase in $T^{*}$ for y>0 is a relaxational effect that is expected to be observed when the value of the relaxation time is equal to, or larger than, the characteristic time of the evolution. Thus, in this specific model, we are able to detect the appearance of a thermal effect directly related to the relaxation time and the vanishing of the asymmetry factor. Before ending this point, it is worth mentioning that we have resorted to the truncated version of the transport equation in order to calculate the temperature. We should recall that such an approximation is valid under certain physical conditions. However, we have not verified these conditions for our model since, as mentioned before, we do not intend to describe a specific astrophysical scenario. It is clear that when dealing with this latter case, the employed transport equation must be properly adapted to the problem under consideration.

Thus, we may conclude that the instantaneously appearance of a TKV produces important effects on the subsequent evolution of the system, some of which are directly related to observable variables (e.g., luminosity and the gravitational redshift). This points to potential applications of the asymmetry factor approach in the study of self-gravitating systems. More specifically, it paves the way to explain some observational astrophysical or cosmological data, which appears unintelligible based on the initial data alone, by resorting to a general approach based on the asymmetry factor. In other words, the asymmetry factor approach seems to be particularly suitable for describing self-gravitating fluid distributions exhibiting an unexpected behavior at some point of its evolution. However, for a full justification of the method, we should be able to identify a physical reason for the vanishing of the asymmetry factor.

We would like to conclude with the following comment. While it is true that specific imprints of the instantaneous appearance of a TKV are clearly model-dependent, the very fact that such an appearance drastically changes the subsequent evolution of the system is not. In addition, for some variables, there seems to exist a persistent imprint for different models (e.g., the complexity factor and the anisotropic factor).

## Data Availability

The original contributions presented in this study are included in the article.

## Appendix A. Einstein Equations

Einstein’s field equations for the interior spacetime (1) are given by

(A1) $$G_{\alpha \beta }=8\pi T_{\alpha \beta },$$

and its non-zero components with (1) and (2) become

(A2) $$\begin{array}{c} 8\pi T_{00}=8\pi \mu =\left(2\frac{\dot{B}}{B}+\frac{\dot{R}}{R}\right)\frac{\dot{R}}{R}-{\left(\frac{1}{B}\right)}^{2}\left[2\frac{R^{\prime \prime }}{R}+{\left(\frac{R^{\prime }}{R}\right)}^{2}-2\frac{B^{\prime }}{B}\frac{R^{\prime }}{R}-{\left(\frac{B}{R}\right)}^{2}\right], \end{array}$$

(A3) $$\begin{array}{c} 8\pi T_{01}=-8\pi qB=-2\left(\frac{{\dot{R}}^{\prime }}{R}-\frac{\dot{B}}{B}\frac{R^{\prime }}{R}\right), \end{array}$$

(A4) $$8\pi T_{11}=8\pi P_{r}B^{2}=-B^{2}\left[2\frac{\ddot{R}}{R}+{\left(\frac{\dot{R}}{R}\right)}^{2}\right]+{\left(\frac{R^{\prime }}{R}\right)}^{2}-{\left(\frac{B}{R}\right)}^{2},$$

(A5) $$\begin{array}{c} 8\pi T_{22}=\frac{8\pi }{\sin^{2}\theta }T_{33}=8\pi P_{⊥}R^{2}=-R^{2}\left[\frac{\ddot{B}}{B}+\frac{\ddot{R}}{R}+\frac{\dot{B}}{B}\frac{\dot{R}}{R}\right]\\ +{\left(\frac{R}{B}\right)}^{2}\left[\frac{R^{\prime \prime }}{R}-\frac{B^{\prime }}{B}\frac{R^{\prime }}{R}\right]. \end{array}$$

The component (A3) can be rewritten with (8) and (10) as

(A6) $$4\pi qB=\frac{1}{3}{\left(\Theta -\sigma \right)}^{\prime }-\sigma \frac{R^{\prime }}{R}.$$
